# Enriched transcriptome analysis of laser capture microdissected populations of single cells to investigate intracellular heterogeneity in immunostained FFPE sections

**DOI:** 10.1016/j.csbj.2021.09.010

**Published:** 2021-09-14

**Authors:** Sarah M. Hammoudeh, Arabella M. Hammoudeh, Thenmozhi Venkatachalam, Surendra Rawat, Manju N. Jayakumar, Mohamed Rahmani, Rifat Hamoudi

**Affiliations:** a College of Medicine, University of Sharjah, Sharjah 27272, United Arab Emirates; b Sharjah Institute for Medical Research, University of Sharjah, Sharjah 27272, United Arab Emirates; c General Surgery Department, Tawam Hospital, SEHA, Al-Ain 15258, United Arab Emirates; d Division of Surgery and Interventional Science, University College London, London, United Kingdom; e Department of Molecular Biology and Genetics, College of Medicine and Health Sciences, Khalifa University, Abu Dhabi, United Arab Emirates

**Keywords:** Intracellular heterogeneity, RNA sequencing, Breast cancer, Immunocytochemistry, Progesterone receptor, NF-κB signaling

## Abstract

To investigate intracellular heterogeneity, cell capture of particular cell populations followed by transcriptome analysis has been highly effective in freshly isolated tissues. However, this approach has been quite challenging in immunostained formalin-fixed paraffin-embedded (FFPE) sections. This study aimed at combining the standard pathology techniques, immunostaining and laser capture microdissection, with whole RNA-sequencing and bioinformatics analysis to characterize FFPE breast cancer cell populations with heterogeneous expression of progesterone receptor (PR). Immunocytochemical analysis revealed that 60% of MCF-7 cells admixture highly express PR. Immunocytochemistry-based targeted RNA-seq (ICC-RNAseq) and in silico functional analysis revealed that the PR-high cell population is associated with upregulation in transcripts implicated in immunomodulatory and inflammatory pathways (e.g. NF-κB and interferon signaling). In contrast, the PR-low cell population is associated with upregulation of genes involved in metabolism and mitochondrial processes as well as EGFR and MAPK signaling. These findings were cross-validated and confirmed in FACS-sorted PR high and PR-low MCF-7 cells and in MDA-MB-231 cells ectopically overexpressing PR. Significantly, ICC-RNAseq could be extended to analyze samples captured at specific spatio-temporal states to investigate gene expression profiles using diverse biomarkers. This would also facilitate our understanding of cell population-specific molecular events driving cancer and potentially other diseases.

## Graphical abstract

**Figure:**
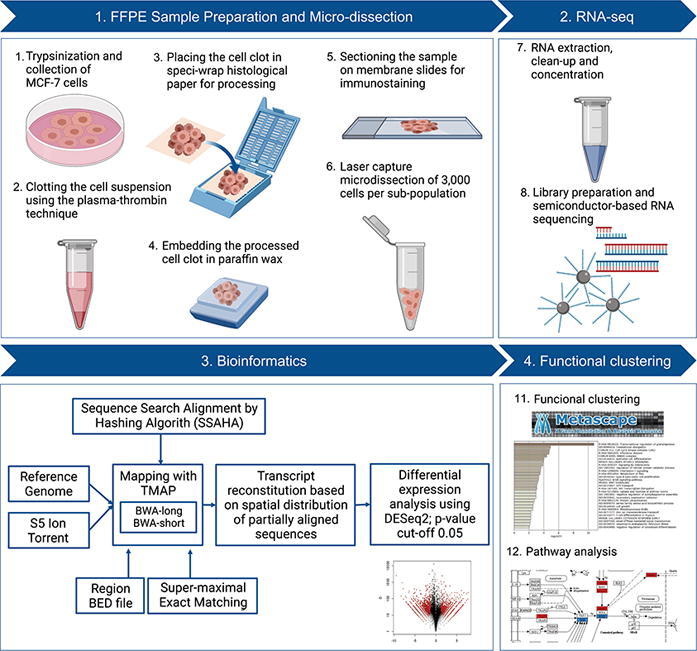

## 1 Introduction

Despite their hormone-dependent origin, breast cancer cells evolve to overcome this dependence resulting in patient resistance to endocrine therapy [1]. However, the evolution process of breast cancer cells varies amongst patients, due to acquired mutations, epigenetic changes, and the diversity of normal and malignant cells forming the tumors. Signs of intra-tumoral heterogeneity were realized at an early stage by J. Huxley in 1958 as he attempted to classify tumors according to their genetic, taxonomic, intra-specific, epigenetic, and environmental heterogeneity status [2].

Recent advances in technologies (e.g. next generation sequencing) permitted more thorough investigations and understanding of tumor heterogeneity [3]. Tumor heterogeneity is currently classified into inter-tumoral heterogeneity (occurring amongst tumors from different patients) and intra-tumoral heterogeneity (occurring between cellular clusters within a single mass) [4]. The heterogeneous cell populations observed within tumors acquire distinct morphologic and phenotypic patterns as a result of various cellular mechanisms including genetic alterations, adaptive transcriptional shifts, and stochastic fluctuations in protein expression [5], [6], [7]. As a result of the complexity introduced by intra-tumoral heterogeneity, various challenges arise including increased cancer aggressiveness, therapy failure and development of drug resistance [8].

The central role of tumor heterogeneity in cancer progression and response to treatment emphasizes the need for increasing the resolution of investigations. Consequently, rapid advances have been achieved in heterogeneous cells capture (e.g. Laser-capture microdissection (LCM), flow sorting, fluidigm C1 microfluidics system, Cyto-seq, and DEPArray) and multi-omics techniques [9], [10]. While these techniques are highly effective in freshly isolated tissues, many of them are incompatible with Formalin Fixed Paraffin Embedded (FFPE) samples, which is the gold standard for long term preservation in histopathology. Therefore, as many of these approaches require cells in suspension, the reproducibility of samples at identical spatio-temporal states is relatively low due to the dynamic nature of cellular mechanisms.

Moreover, capturing and sequencing the RNA content of heterogeneous cell populations in immunostained FFPE sections has been quite challenging. Some studies reported combining protein-based targeting of cell populations using cell sorting systems (e.g. DEPArray system) with whole genome sequencing [11]. However, as DNA from FFPE samples is comparatively more intact than RNA, combining these cell sorting systems with RNA-seq in FFPE samples remains a challenge. On the other hand, proposed approaches that eliminate the need for cell sorters through the use of laser capture microdissection were either applied to frozen samples [12] or histochemically stained (e.g. Cresyl Fast Violet) FFPE sections rather than immunostained FFPE sections [13]. Moreover, approaches that analyze immunostained FFPE sections were combined with targeted gene expression analysis (e.g. qRT-PCR) [14] or genome sequencing [15] rather than RNA-sequencing. In addition, previously proposed approaches combining LCM with RNA-seq isolate regions with heterogeneous phenotypic profiles rather than populations of single cells with heterogeneous expression of biomarkers.

To overcome these limitations, this study combines standard pathology techniques using immunostaining and LCM, with semiconductor-based RNA sequencing [16] and bioinformatics analysis for the targeted selection and characterization of phenotypically heterogeneous cell populations. Using this immunocytochemistry (ICC)-based targeted RNA-seq (ICC-RNAseq) approach, heterogeneous cell populations can be analyzed in cell lines and cellular suspension admixtures. This approach offers the possibility to analyze specific cell populations with high purity based on the distribution and intensity of specific biomarkers and tissue architecture, at cost-effective manner.

This study aimed at using ICC-RNAseq to investigate the molecular implications of progesterone receptor (PR) negative conversion in hormone receptor positive Luminal A breast cancer, the most frequent breast cancer subtype among women [17]. The loss of hormonal receptors, including PR, has been frequently reported in breast cancer patients following the exposure to neoadjuvant therapy (e.g. doxorubicin, cyclophosphamide, docetaxel, and trastuzumab) [18]. Unfortunately, this negative conversion of PR expression has been associated with poor prognosis and shorter survival [18]. However, on the contrary to estrogen receptor and HER2, the molecular implications of PR in the pathogenesis of breast cancer are yet to be extensively investigated. A well profiled cell line model of the luminal A breast cancer, characterized to be positive for the expression of estrogen and progesterone receptors. Intracellular heterogeneity has been reported in the expression of PR in MCF-7 breast cancer cells [19], [20]; however, there is a lack of a comprehensive understanding of the molecular implications of the intracellular heterogeneity in PR expression in cells from the same culture admixture. Therefore, the ICC-RNAseq approach was implemented in this study to investigate the transcriptional and functional implications of the intracellular heterogeneity in progesterone receptor (PR) expression in MCF-7 breast cancer cell line.

## 2 Materials and methods

### 2.1 Processing and staining of MCF-7 blocks

MCF-7 cells were cultured in RPMI supplemented with 10% FBS (Sigma) and 1% penicillin–streptomycin (Sigma) at 37 °C, 5% CO2. The cells were trypsinized with 0.05% Trypsin-EDTA (Sigma), pelleted, and re-suspended in 120 μl human plasma (Sigma) (filtered through 0.2 μM filter). The cell suspension was clotted through the addition of 80 μl of human thrombin (Sigma, dissolved in PBS). The clotted cells were transferred to speci-wrap histological paper (Pioneer Research Chemicals), fixed in formalin for 4 h and processed using the Excelsior AS tissue processor (Thermo Scientific; applied processing protocol in Table S1). Three samples were prepared from three different MCF-7 cell passages.

### 2.2 Immunostaining of paraffin embedded MCF-7 cells

3 µm MCF-7 FFPE sections were adhered to positively charged slides and membrane slides (Leica; coated with 0.01% Poly-L-Lysine) by incubating at 37 °C overnight. Following deparaffinization, the sections were stained using the standard hematoxylin and eosin staining and immunocytochemically for PR, ER and HER2 expression. The immunocytochemical detection of the protein targets was performed using the primary antibodies, ER (Abcam; AB108398 rabbit monoclonal; clone EPR4097; 1:250), PR (Abcam; AB2765 mouse monoclonal; clone Alpha PR6; 1:20) and HER2 (Abcam; AB214275 rabbit monoclonal; clone EPR19547-12; 1:4000). Antigen retrieval was done in pH 9.0 Tris-EDTA solution for ER and Her2 and pH 6.0 sodium citrate solution for PR in microwave oven at 95 °C for 15 min. The primed proteins were chromogenically detected using Ventana’s Optiview DAB IHC Detection kit.

### 2.3 Sections imaging

Images of the stained MCF-7 sections were acquired with Olympus BX43 microscope equipped with Olympus DP75 camera (resolution of 5760 × 3600 pixels and pixel size of 5.86 × 5.86 µm) with Cell Sens Entry software (version 1.17). Images were acquired with 20X (numerical aperture 0.45; 1920 × 1200 pixels; 465.079 nm/pixel resolution in both X and Y axis) and 40X (numerical aperture 0.6; 1920 × 1200 pixels; 232.54 nm/pixel resolution in both X and Y axis) objective lenses.

### 2.4 Manual counting

The cells were counted using the cell counter plugin in ImageJ. A grid was added to the images using the Grid function in ImageJ. Two counters were used for stained/brown cells (red) and non-stained/blue cells (yellow). The cells were counted by two individuals independently, double-checked two days subsequently and averaged to get the final counts. Percentages of stained cells were calculated and compared across three, collectively representative, locations of each section (one representative section stained against ER and one representative section stained against PR) (densely packed areas, areas nearing the edge, and less densely packed areas) (Fig. S1). Manual cell count results are presented as mean ± standard error of the counts from the three representative areas.

### 2.5 Semi-automatic counting

Semi-automatic counting of the heterogeneous cell populations in the MCF-7 section images was performed using ImageJ (version 1.51) using the approach described in [21]. The cell counts were extrapolated from images captured at 20X magnification (.tiff format; size of 1920 × 1200) (Fig. S2a). Initially, we isolated the target population (i.e. stained ER/PR-high and non-stained ER/PR-low cells) using the color binning function in the IHC tool box plug-in (Fig. S2b,c) [22]. We then converted the output images containing either subtype to their binary format through 16-bit conversion and grey scale thresholding (threshold between 100 and 135) (Fig. S2d). We analyzed the cell content of the images using the particle analysis tool to extrapolate the final cell counts. The general workflow for the semi-automatic counting process is described in Fig. S2e. Semi-automatic cell count results are presented as mean ± standard error of the counts from the three representative areas.

### 2.6 Laser capture microdissection

Target cell populations (i.e. PR-high and PR-low cells) were microdissected using Leica LMD 6 Laser Capture Microdissection system (Leica Microsystems CMS GmbH) equipped by Leica’s DFC7000 T camera (resolution of 1920 × 1440 pixels and pixel size of 4.54 × 4.54 µm). The cells were microdissected from immunocytochemically stained MCF-7 FFPE sections adhered to 2.0 µm PEN-membrane slides (Leica). Single cells were dissected at 63X magnification (HCX PL FLUOTAR 63X lens, numerical aperture 0.7). The laser beam settings were adjusted to power 20, aperture 1, and speed 25. 2000–3000 cells were collected from each target population onto the caps of 0.2 ml PCR tubes and preserved at room temperature until extraction. One sample of 2000 cells was collected from each population for qRT-PCR. Samples for whole transcriptome RNA-seq were microdissected from three biological replicates of MCF-7 FFPE preparations. PR-high and PR-low cells population were microdissected from each MCF-7 FFPE preparation; each population containing 3000 microdissected cells.

Triplicate bulk, unsorted MCF-7 cell line samples were harvested in parallel for bulk RNA-seq analysis to emphasize the difference between the common gene expression averaging in bulk analysis as compared to high-resolution analysis using ICC-RNAseq.

### 2.7 Fluorescence-activated cell sorting (FACS) of MCF-7 cells according to PR expression intensity

Freshly harvested cells were suspended in 1 ml of ice-cold PBS and fixed by the gradual addition of 2 ml ice-cold 100% ethanol. The cells were incubated with the ethanol for 1 h at 4 °C. the cells were then centrifuged at 1400 rpm for 5 min (set centrifugation speed and duration throughout the preparation process) to discard the fixative and followed by 2 washes with 3 ml PBS. The cells were then permeabilized using 0.1% Triton X-100 for 15 min at room temperature. The cells were then washed 3 times with 3 ml of PBS. The cells were then blocked using 1%BSA for 1 h at room temperature, the block was discarded following a centrifugation, and the primary antibody was added to the cells at a dilution of 1:1000 over-night at 4 °C. the cells were then washed 3 times with PBS and incubated with the secondary Goat anti-Mouse IgG (H + L), Alexa Fluor 647 antibody (A-21236; Invitrogen) at a 1:800 dilution for 1 h at room temperature. The cells were then washed 3 times with PBS and stored at 4 °C until flow cytometry analysis. Stained MCF-7 cells were sorted into PR-high and PR-low (6000 cells per population) using BD FACS Aria III flow cytometer (BD Biosciences) and BD FACS Diva software.

### 2.8 RNA extraction and qRT-PCR gene expression analysis

Total RNA was extracted from the microdissected cells and FACS-sorted MCF-7 cells using the RecoverAll Total Nucleic Acid isolation kit (Invitrogen) and cleaned up using the RNA Clean and ConcentratorTM-5 kit (Zymo Research).

The difference in the expression of PR between the 2000 microdissected PR-high and PR-low MCF-7 cells was validated by qRT-PCR with 18S rRNA, GAPDH, and β-actin as housekeeping genes. The geometric mean [23] was used to determine a single value as the housekeeping gene which was used to normalize the relative expression calculation of PR. Gene-specific cDNA was synthesized using the SuperScript® III First Strand Synthesis system for RT PCR (Invitrogen). qPCR was performed in triplicates with the Maxima SYBR Green/ROX qPCR Master Mix (Thermoscientific) using QuantStudioTM 5 Real-Time PCR instrument (Applied biosystems). Gene specific cDNA synthesis and qPCR were performed using the primer sequenced in Table 1. Gene expression results are presented as mean ± standard error of triplicates.

**Table 1:** List of primer designs used in the qRT-PCR validation PR expression in LCM-isolated PR-high and PR-low MCF-7 cells using the geometric mean of the housekeeping genes, 18S rRNA, GAPDH, and β-actin (ACTB).

Gene ID	Forward primer sequence	Reverse primer sequence
PR	CACAAAACCTGACACCTCCA	TTCGAAAACCTGGCAATGAT
18S rRNA	TGACTCAACACGGGAAACC	TCGCTCCACCAACTAAGAAC
GAPDH	CTGACTTCAACAGCGACACC	CCCTGTTGCTGTAGCCAAAT
β-actin (ACTB)	TCGTGCGTGACATTAAGGAG	TCAGGCAGCTCGTAGCTCTTT

### 2.9 Transient transfection of MDA-MB-231 cells with PR plasmid

MDA-MB-231 cell line was cultured in High-glucose Dulbecco’s Modified Eagle’s Medium (Sigma; D6429) supplemented with 10% fetal bovine serum, 1% Penicillin/Streptomycin, and 2 mM L-Glutamine. pcDNA3-PRB was a gift from Elizabeth Wilson (Addgene plasmid # 89130; http://n2t.net/addgene:89130; RRID: Addgene_89130) (178).

MDA-MB-231 cells were transfected with 1 µg of pcDNA-PRB plasmid construct for 24 hours using ViaFect^TM^ Transfection reagent (Promega; E4982) according to manufacturers' instruction. Negative control with prepared by treating the cells with plasmid-free ViaFect reagent. RNA was extracted from cell pellets using PureLink RNA Mini kit (Invitrogen; 12183018A), as per the manufacturer’s instructions. RNA samples were cleaned up from potential DNA contaminations using TURBO DNA-free TM Kit (Invitrogen; AM1907).

### 2.10 Whole transcriptome analysis

1 ng of RNA was analyzed using targeted whole RNA-seq with AmpliSeq whole transcriptome on S5 system (Thermo Fisher Scientific). Barcoded CDNA libraries was prepared using SuperScript VILO cDNA synthesis kit (Invitrogen) and amplified using Ion AmpliSeq transcriptome human gene expression kit (Thermo Fisher Scientific) to prepare the RNA-seq library. The quality of the cDNA libraries was assessed using Taqman library quantitation kit (Applied Biosystems). The libraries were diluted to 100 pM, pooled together, amplified using emulsion PCR on Ion One Touch2 instruments (OT2), and enriched on Ion One Touch ES as per manufacturer’s instructions. RNA-sequencing of the libraries was performed using Ion S5 XL Semiconductor sequencer on Ion 540 Chip (Life Technologies).

### 2.11 Bioinformatics analysis

RNA-seq data analysis was performed using the Ion Torrent Software Suite version 5.5. Following base calling using the Basecaller() function within the Torrent Suite, Torrent Mapping Alignment Program (TMAP) (https://github.com/iontorrent/TMAP) was optimized to align the raw sequencing reads generated by the Ion Torrent sequencing data against reference sequence derived from the hg19 (GRCh37) assembly. The immunostained samples sequence tends to be noisier due the FFPE cross linking and heat exposure resulting in fragmentation of the RNA. The RNA fragmentation generates large number of very short sequences as well as smaller number of longer sequences leading to lower coverage from the sequences. In order to improve the sequence coverage and mapped reads, the sequences were assembled through nested alignment algorithm approach using four different alignment algorithms in the order of; Burrows-Wheeler Aligner (BWA)-short [24], BWA-long [25], Sequence Search and Alignment by Hashing Algorithm (SSAHA) [26] and Super-maximal Exact Matching (SMEM) [27], within the TMAP suite. The main commands to carry out the mapping using TMAP suite are: (tmap index -f sequence.fasta) to index the reference sequence and (tmap mapall –q 50,000 -f sequence.fasta -r reads.fastq -v -Y -u -a 3 -s result.bam -o 0 stage1 map4) to carry out the nested alignments.

Each of the short alignment algorithm generates contigs of certain size which is then passed to the next alignment algorithm generating longer contigs and eventually applying de novo alignment using SMEM algorithm [27]. In addition, this approach was shown to provide around 50% more mapped reads and thus better coverage thereby helping to reconstitute the original sequence as much as possible (Table S2).

Final optimal mapping was then derived using Smith-Waterman algorithm [28] and data was merged based on the nearest contigs. Raw read counts of the targeted genes were extrapolated using samtools (samtools view –c –F 4 –L bed_file bam_file). RNAseq data is normalized using Fragments Per Kilobase Million (FPKM) normalisation [29] prior to quality control check. Differentially expressed gene analysis was performed using DESeq2 for samples collected in triplicates (i.e. laser capture microdissected samples and MDA-MB-231 transfected samples) and a modification of the NOISeq algorithm [30] for samples collected without replicates (i.e. MCF-7 FACS sorted cells). The cut-off chosen for DESeq2 is p-value < 0.05 and for NOISeq is q = 0.8 based on the noise of the samples.

### 2.12 In silico functional analysis

Upregulated and downregulated genes were subject to functional analysis using unsupervised hierarchical clustering based on Gene Ontology analysis. Functional clustering was performed using publicly available functional annotation tool, Metascape [31]. The enriched pathways and functional clusters were sorted according to p-value, and filtered according to their relevance (tissue- and disease-wise) and repetition. Heatmap and bar plot representations of the RNA-seq data were generated using R (version 3.6.0). Gene ontology semantics similarity analysis was performed using the GOSemSim() R package [32].

### 2.13 Statistical analysis

Two-tailed *t*-test was conducted to statistically analyze the significance of the cell count and gene expression data; the significance was taken to be p < 0.05. Pearson’s correlation (r) analysis was used to analyze the correlation of cell counts extrapolated from the three representative areas. all statistical analyses were performed using GraphPad Prism (version 5.01).

## 3 Results

### 3.1 Methodology overview

**Fig. 1:**
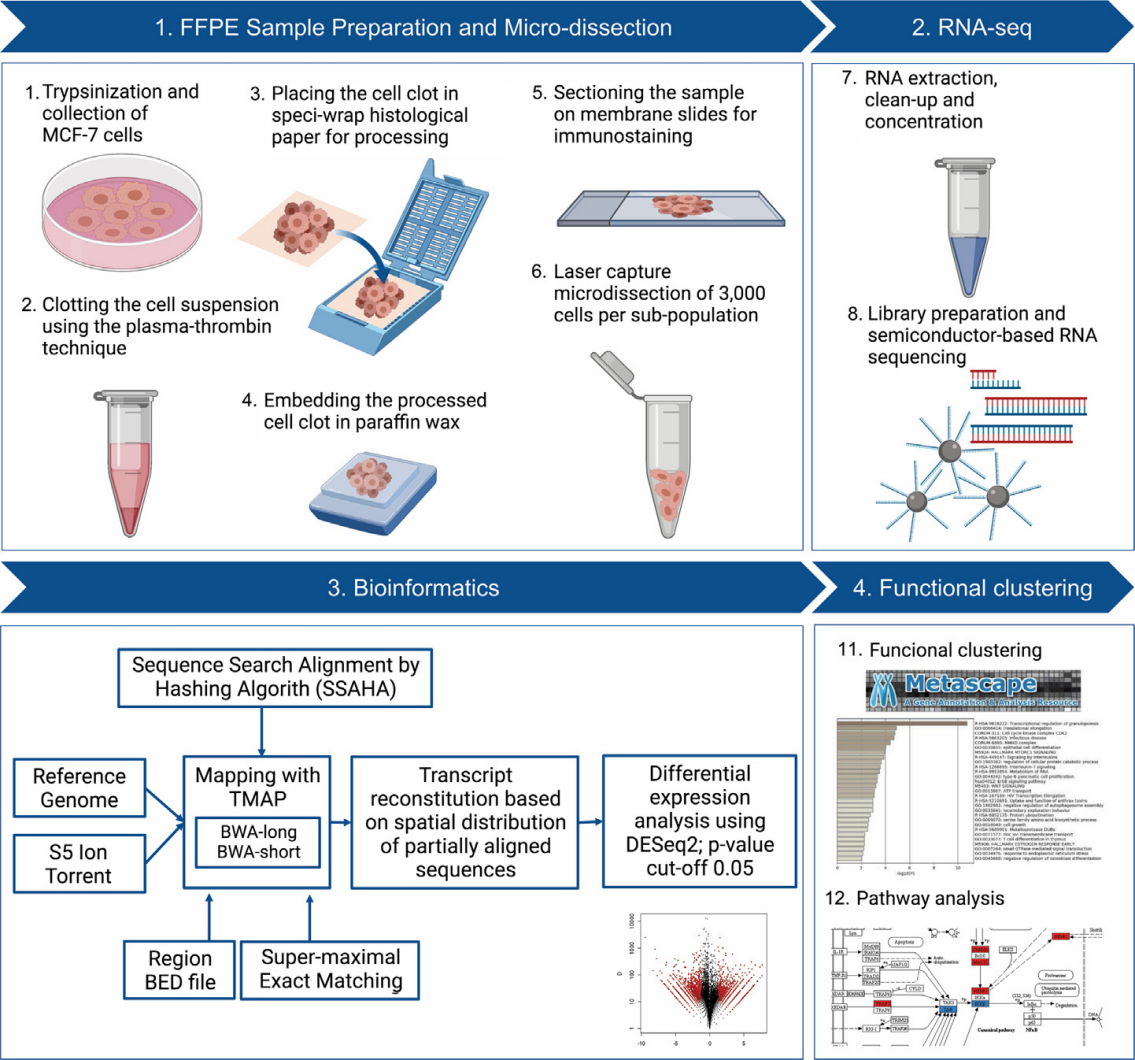
ICC-based targeted RNA-seq (ICC-RNAseq) workflow. Figure was created with BioRender.com

The workflow of ICC-RNAseq integrates histopathology techniques, semiconductor-based RNA sequencing, and bioinformatics analysis to profile the transcriptome of phenotypically heterogeneous cell populations (Fig. 1). In ICC-RNAseq, detached/suspended cells were aggregated using the plasma-thrombin clotting technique for paraffin-embedding [33]. During this cell block generation process, exposure of cells to the plasma-thrombin matrix (∼1 min) was minimized by directly fixing the samples in formalin once the clot is formed. Hence, the effect of human plasma on the cells is minimized in terms of extracellular RNA uptake as well as activation of signaling pathways, which requires ∼6 h incubation with human plasma [34]. In comparison to other cell block preparation approaches, the plasma-thrombin clotting approach is economic, simple, and fast. On the contrary to other types of extracellular matrix (e.g. Matrigel) which induce differential transcriptome due to long term culture [35], the minimal incubation with the plasma-thrombin matrix in this approach overcomes this issue.

The paraffin-embedded cells were sectioned and stained immunocytochemically (ICC) on membrane slides for markers of interest to select the required heterogeneous populations. 3000 cells of each population were laser capture microdissected for RNA extraction, library preparation and semiconductor-based whole transcriptome sequencing. As the cells are microdissecting in a targeted manner, the selective inclusion of cellular bodies and exclusion of surrounding matrix is ensured. Therefore, the possibility of contaminating the samples with extracellular RNA from the surrounding plasma-thrombin matrix is reduced.

The sequencing data is analyzed using a combination of alignment algorithms and the differential transcriptome is derived from the reconstituted transcripts of the heterogeneous cell populations subsequent functional clustering and pathway analysis.

### 3.2 Intracellular heterogeneity in the expression of hormone receptors in MCF-7 breast cancer cells

**Fig. 2:**
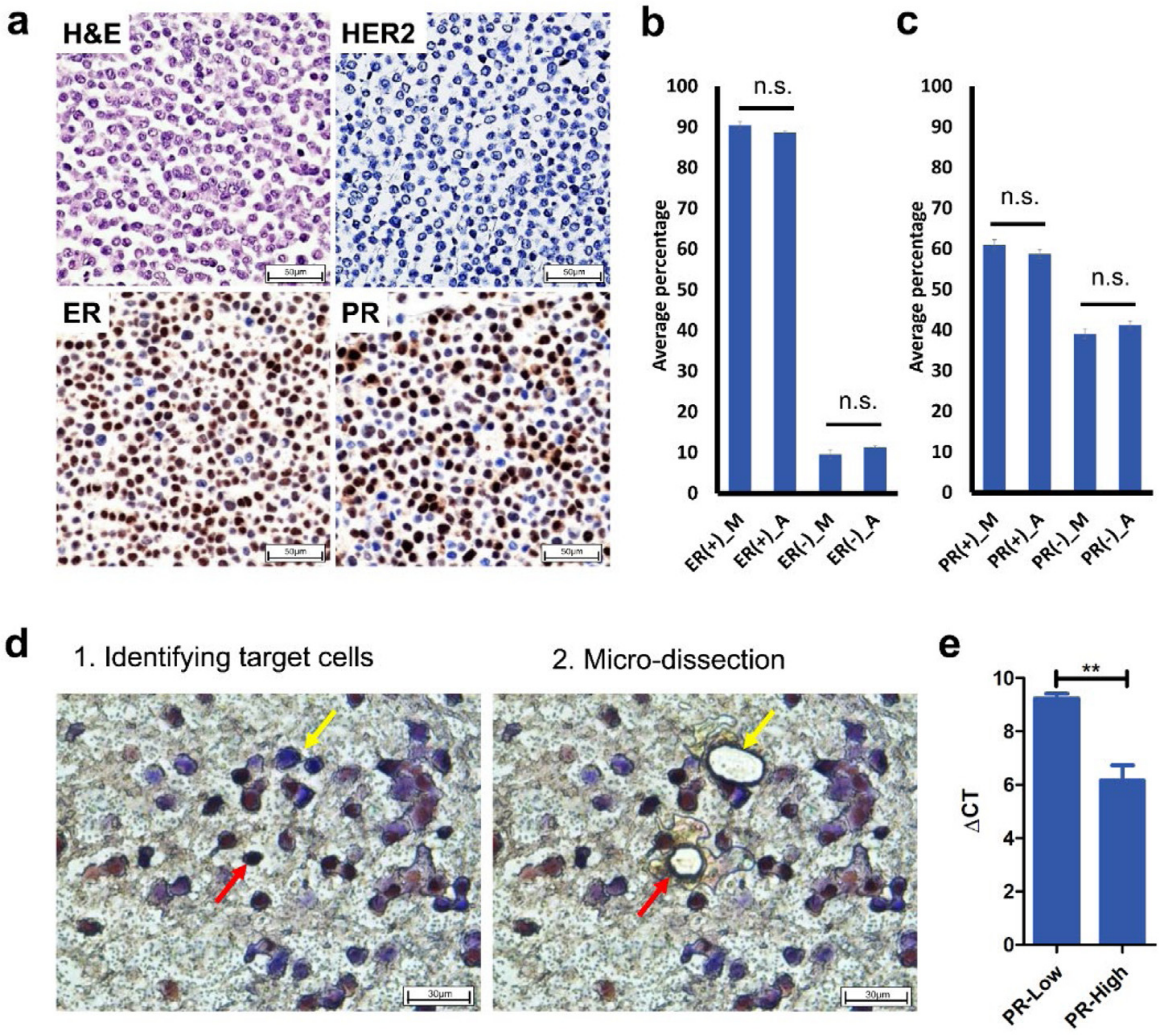
ICC-based identification and isolation of phenotypically heterogeneous cells. (a) chemical (i.e. H&E) and Immunocytochemical characterization of MCF-7 cells according ER, PR, and HER2 expression (images taken at 40X magnification using Olympus BX43 microscope). (b and c) Cell count of cells that have high (ER+/PR+) and low expression (ER-/PR-) of (b) ER and (c) PR extrapolated using manual (M) and semi-automatic (A) counting. (d) Selection of PR positive (red arrow) and PR negative cells (yellow arrow) using laser capture microdissection (Images taken at 63X magnification using Leica LMD 6 Laser Capture Microdissection system). (e) Difference in PR gene expression between PR-high and PR-low microdissected cell using qRT-PCR analysis; PR expression was normalized to the geometric mean of the housekeeping genes 18S rRNA, GAPDH, and β-actin to calculate the ΔCT. Data in the bar plots presented as mean ± SEM. Significance is determined using two-tail *t*-test. (For interpretation of the references to color in this figure legend, the reader is referred to the web version of this article.)

The ICC-RNAseq technique was employed to investigate the intracellular heterogeneity of breast cancer using clinically relevant biomarkers; ER, PR, and HER2, in MCF-7 breast cancer cells. Initially, studies using immunocytochemical staining confirmed that these cells express estrogen receptor (ER) and progesterone receptor (PR), but not Erb-B2 Receptor Tyrosine Kinase 2 (ERBB2; also known as HER2) (Fig. 2a) [36]. Very importantly, the staining revealed a clear heterogeneity in the expression levels of ER and PR.

To quantify the degree of heterogeneity in ER and PR expression, the positive and negative cells were counted manually and semi-automatically in three different representative areas of the MCF-7 sections (Fig. S1). Notably, 89.5 ± 0.6% of cells highly express ER (ER+), while 10.5 ± 0.6% of cells exhibited low expression of ER (ER-) (Fig. 2b). Similarly, only 59.9 ± 0.9% of the cells highly express PR (PR + ), while 40.1 ± 0.9% of the cells express low levels of PR (PR-) (Fig. 2c). The counts extrapolated from the three different areas closely correlated (r = 0.934 for ER+/- cells and r = 0.861 for PR+/-).

### 3.3 Differential expression of PR gene in PR-high and PR-low laser capture microdissected single cell populations

To further validate the differential expression between heterogeneous populations, 2000 immunocytochemically-stained PR-high and PR-low microdissected cells for qRT-PCR analysis (Fig. 2d). PR-high cells had a significantly higher expression of PR at the mRNA level in comparison to PR-low cells (∼8.47 fold change), confirming the differential expression observed at the protein level (Fig. 2e).

### 3.4 Bioinformatics analysis of the whole transcriptome RNA-seq data

To examine the potential implications of the heterogeneous expression of PR, we analyzed the transcriptome of 3000 PR-high and PR-low cells in triplicates using whole transcriptome RNA-sequencing on Ion Torrent S5. To identify differentially expressed genes in PR-high and PR-low cells, gene expression profiling was performed using DESeq2. Notably, 364 genes were differentially upregulated in PR-high cells, whereas 304 genes were differentially upregulated in PR-low cells for a p-value cutoff of 0.05 (Fig. S3a).

To control for the quality of the transcriptome data extracted using the ICC-RNAseq approach, PR expression was extrapolated from the RNA-seq data. PR expression was 10.9-fold higher in PR-high cells in comparison to PR-low cells (Fig. S3b).

### 3.5 PR-high cells are differentially enriched in immunomodulatory NF-kB signaling

**Fig. 3:**
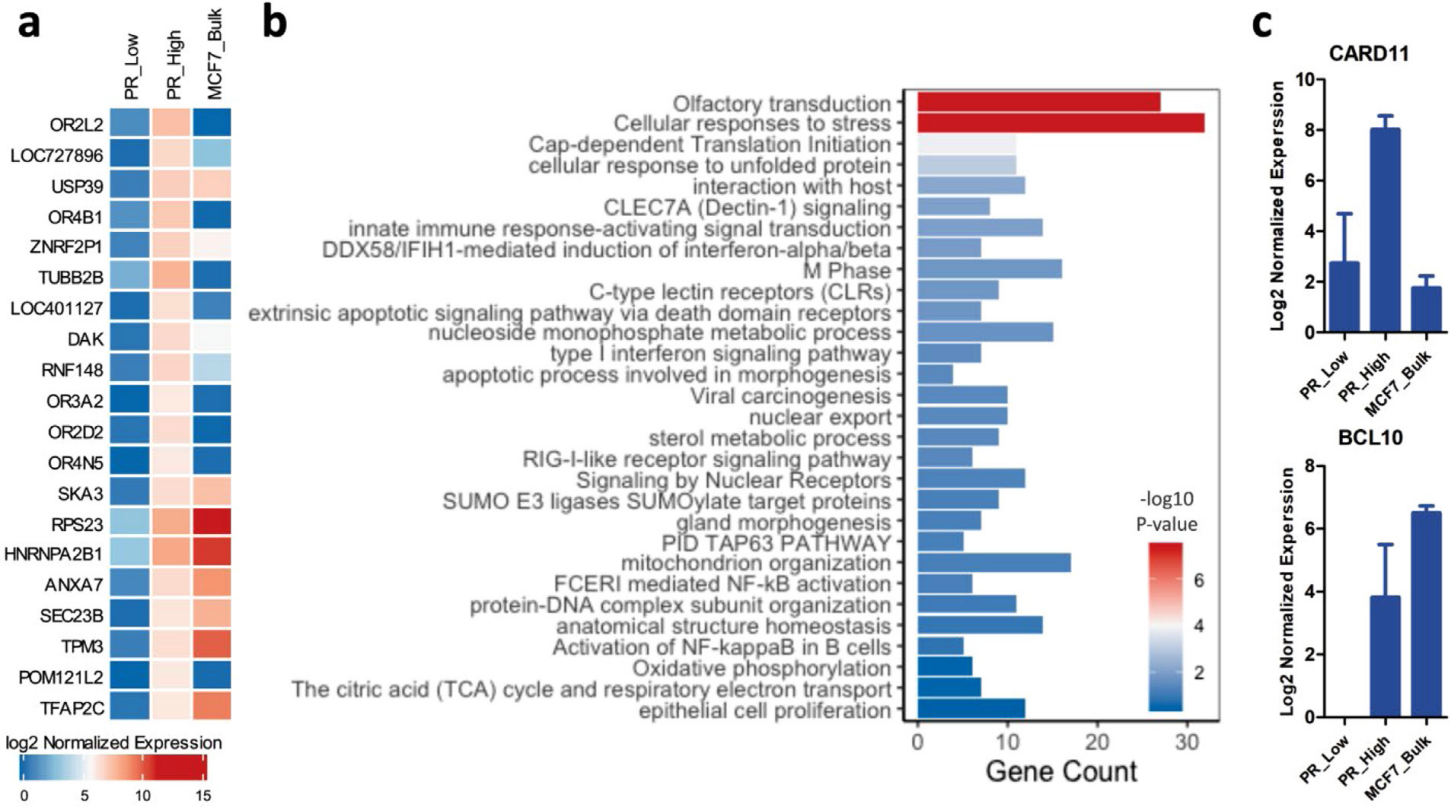
Functional clustering of differential transcriptome in PR-high cells. (a) The top 20 differentially upregulated genes in PR-high cells in comparison to PR-low LCM-isolated MCF-7 cells. Normalized expression of the genes in bulk, unsorted MCF-7 samples was added to elaborate population difference in expression at different resolution levels. The heatmap was generated using ComplexHeatmap () package in R and represents the mean of log2 of normalized gene expression of triplicates analyzed using DESeq2 algorithm; the genes were filtered according to a p-value cut-off of <0.05. (b) The top 30 enriched pathways in PR-high cells extrapolated from functional clustering analysis using Metascape. Barplot was generated using ggplot2 package in R; p-value cut-off for pathways inclusion was <0.01. (c) Difference in genes expression of the CBM-complex molecules CARD11 and BCL10, which are part of NF-κB signaling pathway, between PR-high and PR-low cells observed from RNA-seq data.

The top significantly upregulated genes in PR-high cells (Fig. 3a) included ubiquitin-specific peptidase 39 (USP39), a pre-catalytic spliceosome member; the Spindle and Kinetochore Associated Complex Subunit (SKA3), a regulator of microtubule attachment to the kinetochores during mitosis; and tropomyosin3 (TPM3), an actin-binding protein that stabilizes cytoskeletal microfilaments. These genes have been shown to contribute significantly to enhanced cancer cells proliferation, tumor progression, and metastasis in a number of cancer types including breast cancer [37], [38], [39], [40].

To investigate the functional relevance of the significantly upregulated genes in PR-high cells (364 genes; p-value < 0.05), functional clustering and pathway analysis was performed using Metascape (Fig. 3b). In concordance with the relatively elevated expression of PR in the PR-high MCF-7 cells population, the functional clustering analysis revealed enrichment for signaling by nuclear receptors (Reactome Gene Set R-HSA-9006931) and nucleocytoplasmic transport (GO:0051168, GO:0006913), which may potentially link to the dynamic activation state of PR through its continuous shuttling to the nucleus upon diffusion to the cytoplasm [41]. Consistently, a focused analysis of the steroid hormone mediated signaling gene ontology set (GO:0043401) and nuclear receptor activity (GO:0004879) revealed the dysregulation of a number of genes implicated in the regulation of these pathways including PGR, PHB, BMP7, HEYL, SMARCA4, DDX5, RHOA, LATS1, DDX17, PTGES3, RXRA, YAP1, SRC, NR2F6, NR1I3, RORC, HNF4G, and SREBF1.

Moreover, the functional clustering analysis revealed a significant enrichment for transcripts implicated in regulating downstream signaling of pattern recognition receptors (PRRs) (e.g. C-type lectin receptors, Dectin -1, RIG-I), innate immune response-activating signal transduction, and NF-κB signaling and its target pathways (e.g. Tap63 signaling pathway [42]). Overexpression of PRRs in cancer cells has been shown to have a tumor promoting role by inducing autoregulative tumor cell growth and anti-apoptotic Bcl-xL expression [43]. Furthermore, the modulation of Type I interferon signaling in cancer cells, as observed in the PR-high populations, plays an important role in the immune evasion of cytotoxic T-cells [44].

Intriguingly, PR-high cells were found to differentially express CARD11 and BCL10, members of the CARD-BCL10-MALT1 complex which regulates NF-κB signaling (Fig. 3c). The enrichment of elements of the NF-κB signaling pathway is consistent with previous studies suggesting a regulatory role of PR on NF-κB signaling through interaction with elements of the NF-κB pathway (e.g. p65) and co-recruitment to the promoters of target genes [45], [46], [47]. Moreover, the upregulated genes involved in PRRs and immune receptor signaling pathways shown by the functional clustering analysis (e.g. PSMB4, PSMD8, RPS27A, UBE2D1, BCL10, CARD11, CALM3, PPP3CA) are commonly activated genes by NF-κB pathway.

Consistently, PR-high cells were significantly enriched as well in epithelial cell proliferation, cell cycle, apoptotic pathways that regulated tissue homeostasis and tissue morphogenesis suggesting the potential activation of tissue remodeling mechanisms. Another enriched pathway is the unfolded protein response, an adaptive cellular response to stressful conditions (e.g. reduced oxygen and energy supply) frequently observed in breast cancer [48]. Other enriched pathways included signaling through GPCRs (Olfactory receptors from the rhodopsin-like receptor family), metabolic process, translation, and protein modification.

To show the variance in population-specific gene expression between bulk analysis of unsorted samples in comparison to LCM-isolated PR-High and PR-Low MCF-7 cells, the normalized expression of the top significantly upregulated genes was assessed in bulk, unsorted MCF-7 cell line samples (Fig. 3a, Fig. 3c). The expression of the genes in the unsorted samples differed from the expression of genes in the LCM-sorted MCF-7 cells, confirming that the expression of genes in admixture heterogeneous cell populations is masked when bulk samples are analyzed.

### 3.6 PR-low MCF-7 cells are differentially enriched during mitochondrial metabolic processes as well as in EGFR and MAPK signaling

**Fig. 4:**
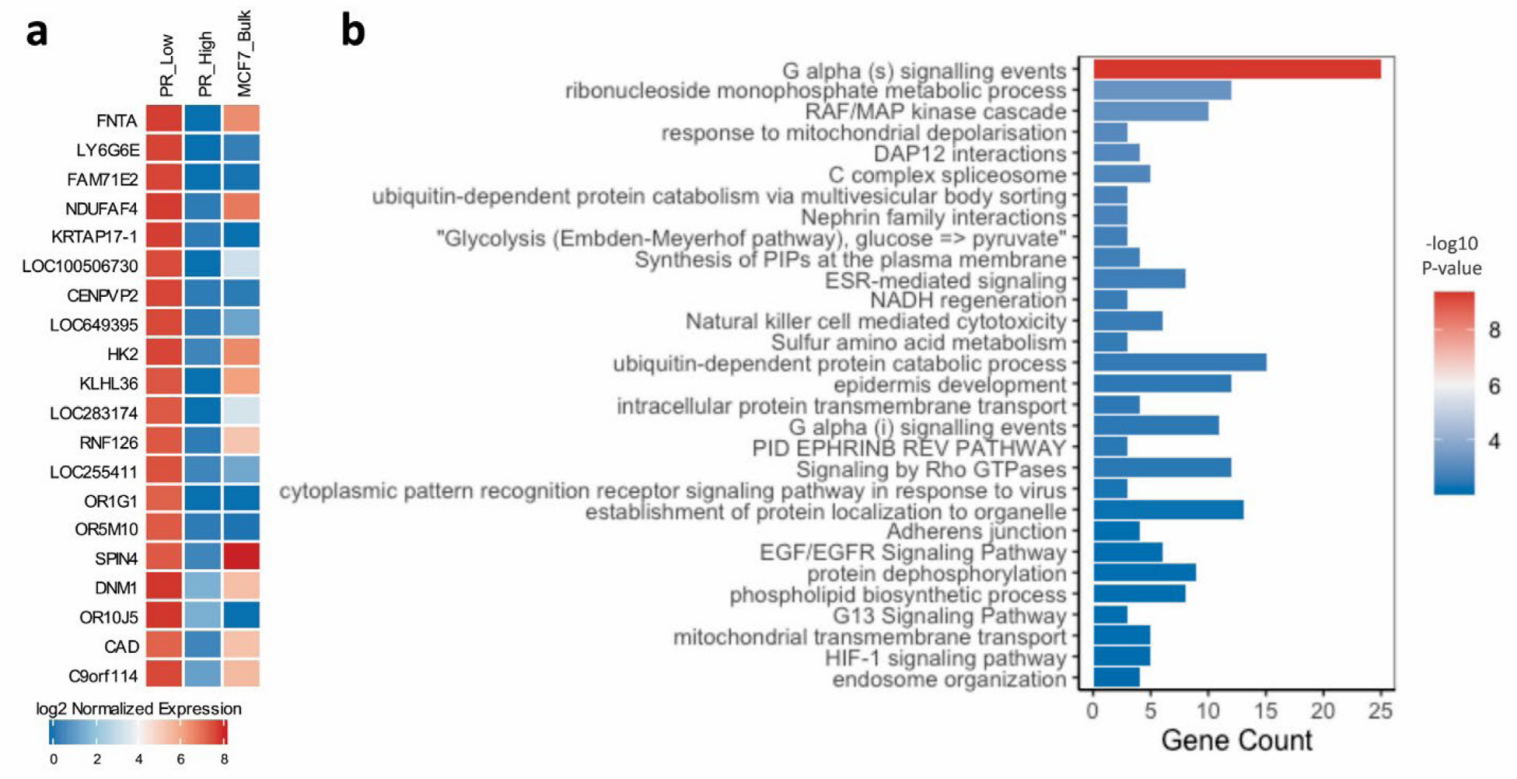
Functional clustering of differential transcriptome in PR-low cells. (a) The top 20 differentially upregulated genes in PR-low cells in comparison to PR-high LCM-isolated MCF-7 cells. Normalized expression of the genes in bulk, unsorted MCF-7 samples was added to elaborate population difference in expression at different resolution levels. The heatmap was generated using ComplexHeatmap () package in R and represents the mean of log2 normalized gene expression of triplicates analyzed using DESeq2 algorithm; the genes were filtered according to a p-value cut-off of <0.05. (b) The top 30 enriched pathways in PR-low cells extrapolated from Metascape functional clustering analysis. Barplot was generated using ggplot2 package in R; p-value cut-off for pathways inclusion was <0.01.

The top significantly upregulated gene in the PR-low cell population (Fig. 4a) is Farnesyltransferase (FNTA), a central regulator of pro-tumorigenic and pro-metastatic GTPases signaling through regulating GTPases translocation to the plasma membrane [49], [50], [51]. Another top upregulated gene in PR-low cells is SPOUT Domain Containing Methyltransferase 1 (C9orf114; also known as SPOUT1), a methyltransferase and kinetochore-associated protein involved chromosomes alignment, mitotic spindle assembly and cell division [52]. Moreover, dynamin 1 (DNM1), another top upregulated gene, was shown to contribute to aberrant vesicular trafficking of receptors and signaling cascades elements to the plasma membrane, thereby altering cell signaling and enhancing cell proliferation [53].

Functional clustering of the differentially upregulated genes in PR-low cells (304 genes; p-value < 0.05) showed an enrichment for various metabolic pathways (e.g. ribonucleoside monophosphate, Sulfur amino acid, proteins, glucose) and biosynthetic pathways (e.g. phospholipid, phosphatidylinositol phosphates) as well as mitochondrial processes (mitochondrial depolarization, NADH regeneration, mitochondrial transmembrane transport).

Moreover, various pro-tumorigenic signaling pathways were activated in PR low cells including MAPK, Rho GTPases, EGFR, ESR, and HIF-1 signaling pathways (Fig. 4b). Upregulation of these pathways has been shown to contribute to hormone receptor positive cancers resistance post adjuvant endocrine treatments [54], [55], [56], [57]. Furthermore, PR low cells are enriched in transcripts involved the regulation of adherens junctions, cell–cell interactions (e.g. Nephrin family interactions, ephrin B1 reverse signaling), and cytoskeletal organization (G13 Signaling Pathway) which might suggest potential morphological shifts in comparison to PR high MCF-7 cells.

Taken together, these findings suggest that MCF-7 cells potentially acquire adaptive transcriptional changes resulting in their phenotypic shift and activation of potential pathways linked to resistance mechanisms. Notably, gene expression profiles observed in the unsorted samples significantly differed from those observed in the LCM-sorted MCF-7 cells, indicating that LCM-sorted single cell population analysis provides deeper insights into the role of the specific biomarkers in the disease whereas the bulk analysis shows admixture expression of genes in PR-high and PR-low expressing cells.

### 3.7 Functional clustering analysis of differentially expressed genes in FACS sorted PR-high and PR-low MCF-7 cells

To further validate our findings from the ICC-RNAseq analysis of the microdissected PR-high and PR-low MCF-7 cells, freshly collected MCF-7 cells were FACS sorted into PR-high and PR-low populations and analyzed using whole transcriptome RNA-seq. their differential transcriptome using bulk RNA-sequencing. Differentially expressed genes were identified using the NOISeq algorithm [30], which can accommodate samples with single replicates as opposed to DESeq2. The cut-off chosen for NOISeq is q = 0.8.

Comparative analysis between the microdissected samples and FACS sorted samples revealed the common upregulation of 183 genes in the PR-high cell populations analyzed using the two approaches. Consistent with the ICC-RNAseq analysis, comparative analysis of genes involved in steroid hormone mediated signaling (GO:0043401) and nuclear receptor activity (GO:0004879) between FACS isolated PR-high and PR-low MCF-7 cells revealed the dysregulation of a number of genes including BMP7, DDX5, RHOA, SMARCA4, LATS1, SREBF1, HNF4G, and RORC. Moreover, the functional clustering analysis revealed a significant enrichment for progesterone and steroid hormone biosynthesis. Taken together, these results confirm a shift in the transcriptional program of pathways directly linked to PR expression and activity.

**Fig. 5:**
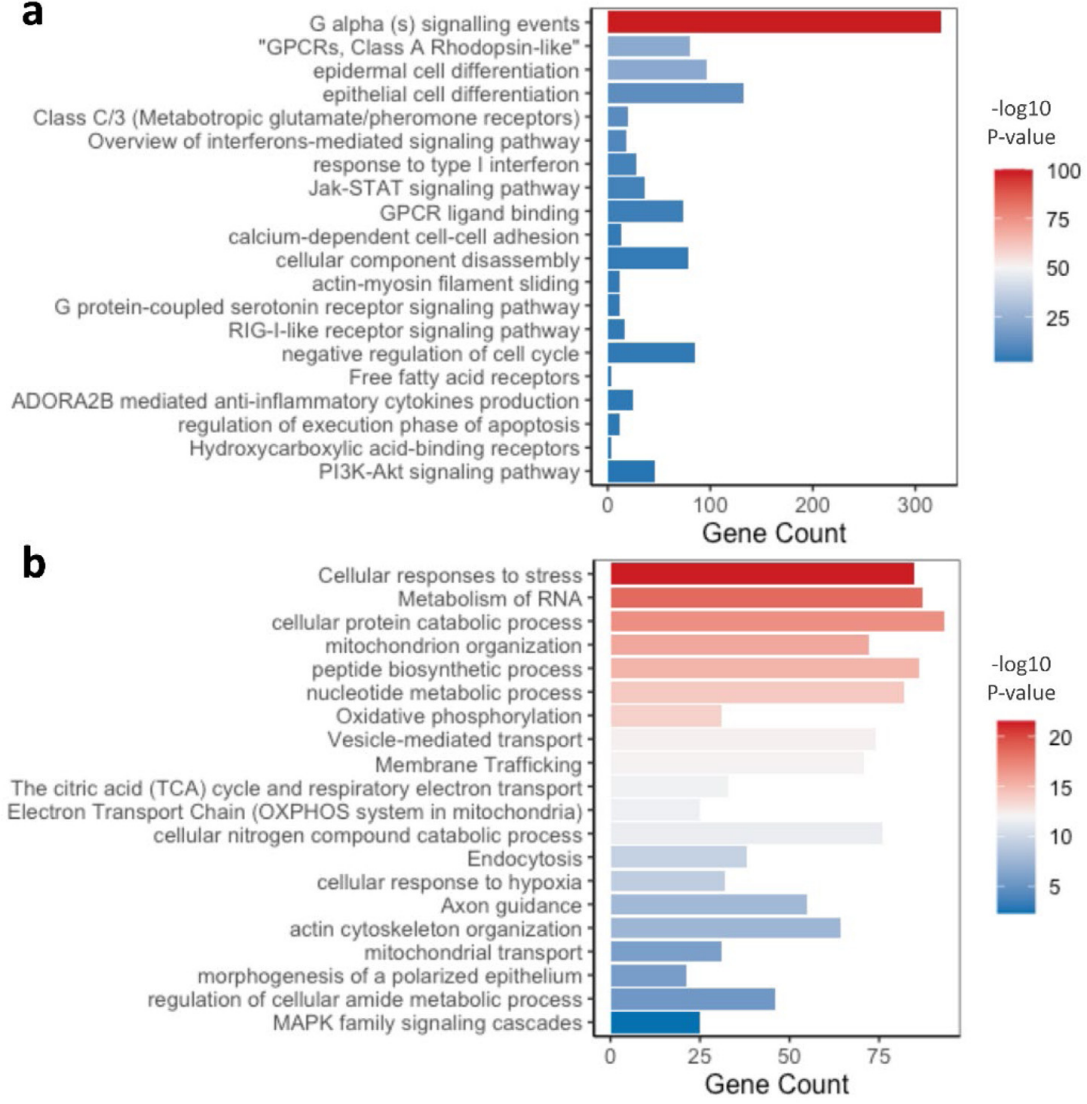
Functional clustering of differential transcriptome in FACS sorted MCF-7 cells according to PR expression. 20 of the top enriched pathways in the differentially upregulated transcriptome of (a) PR-high and (b) PR-low FACS isolated MCF-7 cells. The barplots selectively presents pathways and functional clusters similar to those enriched in the laser capture microdissected PR-high and PR-low MCF-7 cells. Barplots were generated using ggplot2 package in R; p-value cut-off for pathways inclusion was <0.01.

Commonly upregulated genes included a number of interferon expressing genes (IFNA2, IFNA17, IFNE) as well as the IRF family member IRF3. Functional clustering analysis of these commonly upregulated genes confirmed the significant enrichment for inflammatory pathways including TRAF6 mediated IRF7 activation, interferon signaling, RIG-I-like receptor signaling pathway, JAK-STAT signaling, and B cell and natural killer cells activation, and anti-viral immune response. Furthermore, in correlation with the differential transcriptome of the microdissected PR-high MCF-7 cells, PR-high FACS-sorted cells were significantly enriched in GPCRs signaling, epidermal development, epithelial cell differentiation, and tissue morphogenesis (Fig. 5a).

Gene ontology semantic analysis using GOSemSim() tool revealed a semantic similarity higher than 0.5 in pathways related to GPCRs activity, binding to protein specific domains, heat shock protein, chaperones, cell adhesion molecules, phosphatidylinositol 3-kinase, histone, as well as type I interferon receptor binding, B cell differentiation and proliferation, natural killer cell activation in immune response, JAK-STAT signaling, defense response to viruses and double stranded RNA, steroid biosynthetic process between PR-high LCM-isolated MCF-7 cells and FACS-isolated PR-high MCF-7 cells (Table S3).

**Fig. 6:**
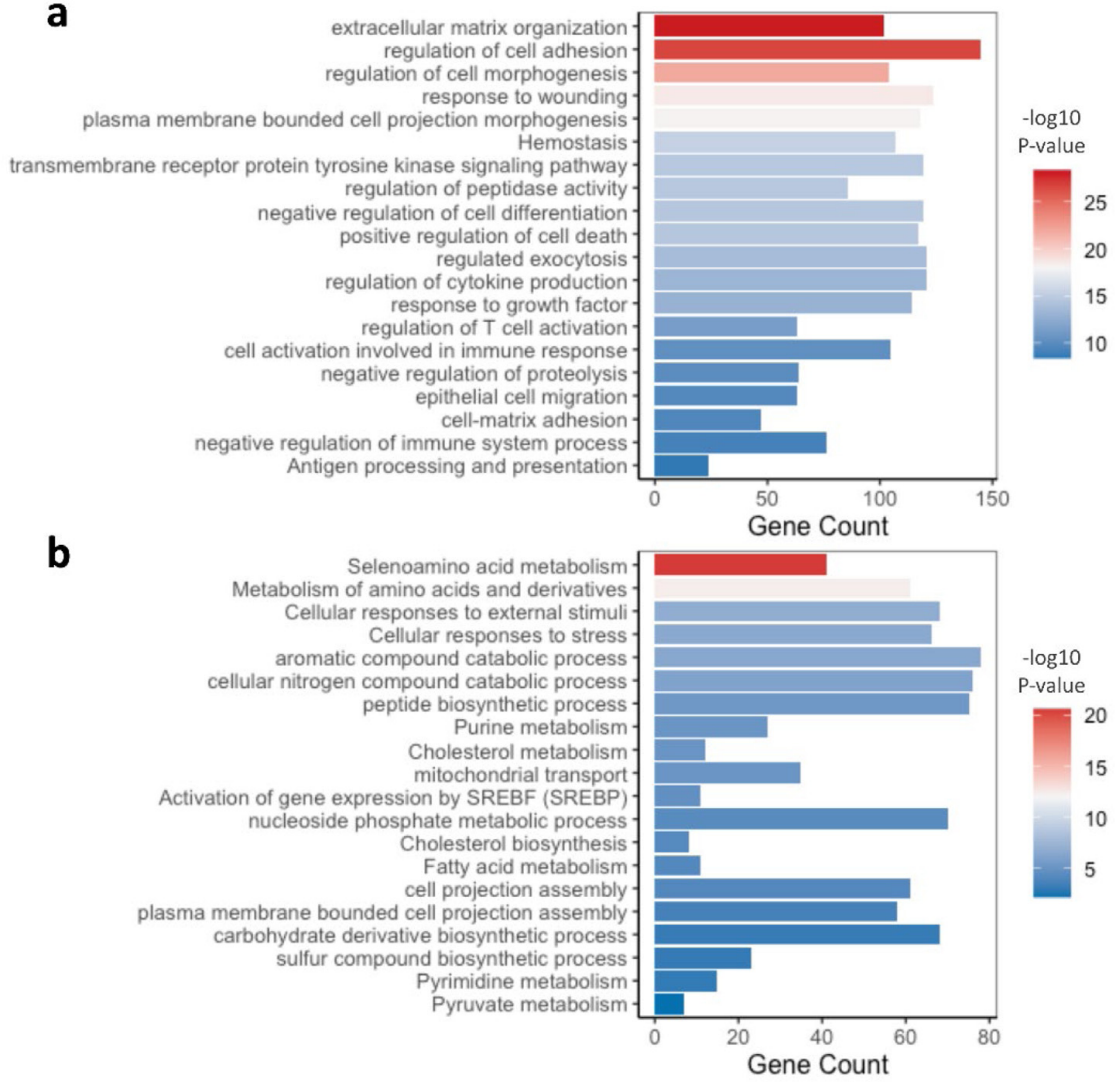
Functional clustering of differential transcriptome in PR-overexpressing MDA-MB-231 cells. 20 of the top enriched pathways in the (a) upregulated and (b) downregulated transcriptome of the PR-overexpressing MDA-MB-231 cells extrapolated from functional clustering analysis using Metascape. The barplots selectively presents pathways and functional clusters similar to those enriched in the laser capture microdissected PR-high and PR-low MCF-7 cells. Barplots were generated using ggplot2 package in R; p-value cut-off for pathways inclusion was <0.01.

On the other hand, FACS-sorted PR-low cells were consistently enriched in metabolic and synthetic pathways, mitochondrial processes (Oxidative phosphorylation, respiratory chain reaction, citric acid cycle and electron transport chain), intracellular transport, cytoskeleton organization, MAPK signaling, response to hypoxia (Fig. 6b). Gene ontology semantic analysis confirmed a semantic similarity higher than 0.5 in pathways related to ubiquitin-dependent protein catabolic process, spliceosome catalytic process, establishment of protein localization to mitochondrion, generation of precursor metabolites and energy between PR-low LCM-isolated MCF-7 cells and FACS-isolated PR-low MCF-7 cells (Table S4).

### 3.8 Functional clustering analysis of differentially expressed genes in PR-overexpressing MDA-MB-231 cells

To further confirm the correlation of the observed shifts in functional clusters and pathways to the status of PR expression, PR was overexpressed in the ER/PR-negative MDA-MB-231 breast cancer cell line using transient transfection.

Although MDA-MB-231 cells are genetically and phenotypically distinct and lack the expression of estrogen receptor, an important co-regulator and cross-talk partner of PR; analysis of genes involved in steroid hormone mediated signaling (GO:0043401) and nuclear receptor activity (GO:0004879) in PR-overexpressing MDA-MB-231 cells revealed the dysregulation of a number of genes including HEYL, PGR, SMARCA4, HNF4G, and SREBF1. Therefore, confirming shifts in cellular pathways and processes directly relating to PR activity.

Consistently with the profile of the PR high, PR-overexpressing MDA-MB-231 cells were significantly enriched for transcripts involved in tissue remodeling (ECM organization, cellular morphogenesis, epithelial cells migration, wound healing, and homeostasis), activation and differentiation of immune response effectors, apoptotic pathways, and signaling through GPCRs (transmembrane receptor protein tyrosine kinases and growth factor receptors) (Fig. 6a).

Gene ontology semantic analysis using GOSemSim() tool revealed a semantic similarity higher than 0.5 in pathways related to binding to protein specific domains, chaperones, heat shock proteins, cell adhesion molecules, phosphatidylinositol 3-kinase, histone, and cadherin as well as T cells and lymphocytes activation, lymphocyte and leukocytes proliferation, gland and morphogenesis and development between PR-high LCM-isolated MCF-7 cells and PR-overexpressing MDA-MB-231 cells (Table S5).

On the other hand, consistently with the PR low microdissected cells, the downregulated transcriptome of the PR-transfected MDA-MB-231 cells was significantly enriched in transcripts involved in metabolic and biosynthetic pathways, mitochondrial transmembrane transport, cell projections assembly and cell morphological shifts (Fig. 6b). Gene ontology semantic analysis confirmed a semantic similarity higher than 0.5 in pathways related to metabolic and biosynthetic pathways, olfactory receptor activity and cell cycle regulation between PR-low LCM-isolated MCF-7 cells and the downregulated transcriptome of the PR-overexpressing MDA-MB-231 cells (Table S6).

## 4 Discussion

Despite the rapid development in heterogeneous cells capture and sequencing techniques, effective approaches combining biomarker-based targeted cell selection and RNA-seq in FFPE samples are needed. This study presents the ICC-RNAseq approach, a user-friendly targeted selection approach of phenotypically characterized heterogeneous cells from FFPE samples for transcriptome analysis.

The selection of the heterogeneous cell populations is based on the detection of biomarker expression using immunocytochemistry. The high perceptibility of the chromogenic labelling of biomarkers enhances the purity of the collected samples using laser capture microdissection. However, due to the exposure of these samples to harsh conditions (e.g. heat and chemical treatments), it is expected that their RNA content would be severely degraded. Therefore, this approach applies an adaptive alignment approach that combines various alignment algorithms suitable for different amplicon sizes and reconstitutes the partially aligned sequences based on spatial distribution. The use of the LCM for the selection of the heterogeneous populations eliminates the need for sophisticated cell sorters not readily available for using in every facility. Moreover, as the samples are fixed, subsequent priming with the antibodies will not affect the transcriptome of the cells. This approach was used to extract the differential transcriptomes of the selected heterogeneous cell populations in this study. The methodology presented can be extended to facilitate our understanding of cell population-specific molecular events driving diverse intracellularly heterogeneous diseases.

The loss of hormonal receptors, including PR, has been frequently reported in breast cancer patients in response to exposure to therapy, which unfortunately associates with poor prognosis and shorter survival [18]. Consistently, heterogeneous negative conversion of PR has been reported in the MCF-7 luminal breast cancer cell line model [19], [20]. In this study, ICC-RNAseq was employed to investigate the transcriptional implications of the reported intracellular heterogeneity in PR expression within MCF-7, a hormone receptor positive breast cancer cell line [19], [20].

Our findings showed that approximately 60% of MCF-7 cells highly express PR, while the remaining cells present with a significantly lower expression of PR. Similarly, ∼90% of the cells display high expression of ER. The results of this study provided an evidence of intracellular heterogeneity in biomarker expression amongst genetically identical cells and proposed an approach to quantify this intracellular heterogeneity. However, a potential limitation of the ICC-based selection of the cells is the possibility of false positive selection of cells to which the antibody bound non-specifically.

RNA-seq analysis of the PR high and PR low cell populations confirmed substantial changes in the activated signaling pathways in correlation with PR expression levels. Our findings suggest that the PR high cell population acquired an immune-modulatory phenotype in which NF-κB signaling pathway is highly upregulated. Moreover, this study revealed the upregulation of the NF-κB signaling regulatory CBM complex proteins, CARD11 and BCL10, in correlation with higher expression of PR. These results are substantiated by previous studies suggesting direct regulatory interactions between PR and NF-κB signaling elements [45], [46], [47]. Through interaction and co-recruitment to the promoter sights of NF-κB inducible genes, PR exerts an immunomodulatory effect in breast cancer [45], [58]. However, while NF-κB signaling is a major modulatory mechanism of immune response, it also plays a central role in cancer cells survival and proliferation. Concordantly, immunomodulatory pathways and inflammatory mediators have been reported to be activated in breast cancer to promote tumor progression through the activation of NF-κB signaling [59]. Therefore, the activation of these immunomodulatory pathways we observe in PR-high cells might be a pro-tumorigenic mechanism to shift cellular signaling towards survival, growth, and proliferation.

Alternatively, our analysis revealed that the PR low MCF-7 cell population was enriched for EGFR and MAPK signaling, indicating a potential compensatory transcriptional shift in response to the loss of PR expression. Consistently with these results, previous studies showed that the loss of PR expression in originally hormone receptor (ER and PR) positive breast cancer cells was compensated by an increase in EGFR signaling, resulting in the acquisition of resistance to selective ER modulators (SERM; e.g. tamoxifen) [60], [61]. Our analysis suggested as well that the suppression of PR expression might influence metabolic and biosynthetic pathways and mitochondrial function suggesting a potential role of PR in the regulation of mitochondrial processes. These findings are consistent with previous studies which proposed that progesterone stimulates mitochondrial activity in breast epithelial cells [62].

Notably, changes in the pathways identified using ICC-RNAseq were confirmed by studies using FACS isolated PR-high and PR-low MCF-7 cells as well as MDA-MB-231 cells ectopically overexpressing PR. Whilst this study cross-validated the findings from the ICC-RNAseq approach, a main limitation of this study is the lack of further functional and mechanistic studies to confirm the direct association between PR expression and deregulation of the identified signaling pathways; which is to be addressed in future studies. Moreover, although microdissection can permit the selection of cells with similar degrees of PR positivity, cells are still likely to have variations in the expression of PR and other biomarkers. Therefore, it is important to consider these additional layers of heterogeneity within the isolated cell populations. Moreover, the trypsinization process employed to harvest the cells can disrupt the plasma membrane and change its constitution [63], which might distort the detection of surface markers expression.

ICC-RNAseq is a flexible approach that can be applied to investigate pathological mechanisms using target biomarkers in cell lines and cellular suspensions. ICC-RNAseq provides a preserved snapshot of the sample that can be used for the analysis of diverse biomarkers from low starting amount over a prolonged period of time. Therefore, ICC-RNAseq can be used to recursively track and identify signaling molecules in target pathways, by reiteratively identifying biomarkers from ICC-RNAseq and microdissecting populations positively expressing these biomarkers for subsequent ICC-RNAseq analysis. This can help to elucidate the molecular mechanism of disease by identifying molecules which exist at a specific time points in the cell life cycle. However, this application would require continuous repetition of ICC, LCM microdissection and RNA-seq.

Moreover, ICC-RNAseq can potentially be adapted in future work to investigate heterogeneous cell populations in patient tissues samples, while correlating biomarker distribution and intensity of expression with tissue architecture. Additionally, visual access to the stained cells can enhance the purity of the selected cells according to the required intensity of biomarker expression. In summary, ICC-RNAseq can be utilized to investigate various aspects of intracellular heterogeneity including heterogeneous response to therapeutic treatment as well as adaptive transcriptional shifts based on external triggers which is not possible to study in bulk analysis.

## 5 Conclusion

In conclusion, the study presented an ICC-based targeted approach that combines routine pathology techniques, immunocytochemistry and LCM, with semiconductor-based RNA sequencing and modified bioinformatics approaches for the targeted selection and characterization of phenotypically heterogeneous cell populations. Using this approach, characterization of MCF-7 single cells populations heterogeneously expressing PR was carried out. The results indicated that the loss of PR expression results in the shift of the cellular transcriptional profile resulting in compensatory intracellular signaling and metabolic modulation.

## 6 Data Availability Statement

RNA-seq datasets for laser capture microdissected MCF-7 samples, FACS sorted MCF-7 cells, and PR-transfected MDA-MB-231 cells samples as well as the modified NOISeq script used in study analysis filtering are supplied as supplementary materials.

## Funding

This research was funded by Al-Jalila Foundation, grant code AJF201741; Sharjah Research Academy, grant code MED001; University of Sharjah, grant code 1901090254 and Sheikh Hamdan Award for Medical Science Research (Grant No: MRG/108/2018).

## CRediT authorship contribution statement

**Sarah M. Hammoudeh:** Conceptualization, Methodology, Software, Investigation, Validation, Formal analysis, Writing – original draft, Writing – review & editing, Visualization. **Arabella M. Hammoudeh:** Investigation, Validation, Writing – original draft, Writing – review & editing. **Thenmozhi Venkatachalam:** Investigation, Validation, Writing – review & editing. **Surendra Rawat:** Investigation, Validation, Writing – review & editing. **Manju N. Jayakumar:** Investigation, Validation, Writing – review & editing. **Mohamed Rahmani:** Formal analysis, Writing – original draft, Writing – review & editing, Supervision. **Rifat Hamoudi:** Conceptualization, Methodology, Software, Formal analysis, Writing – original draft, Writing – review & editing, Supervision, Funding acquisition.

## Declaration of Competing Interest

The authors declare that they have no known competing financial interests or personal relationships that could have appeared to influence the work reported in this paper.
